# Impact of High-Fat Diet and Aflatoxin B1 on Immunometabolic Dysfunction and the Dose-Responsive Modulation by Isoleucine Supplementation

**DOI:** 10.3390/nu17172897

**Published:** 2025-09-08

**Authors:** Ruojin Wang, Jiangli Wang, Meifang Lan, Xiyin Wang

**Affiliations:** 1 School of Public Health, North China University of Science and Technology, Tangshan 063210, China; m15100539998@163.com (R.W.); 18093830463@163.com (J.W.); 2 State Key Laboratory of Plant Diversity and Specialty Crops and Key Laboratory of Systematic and Evolutionary Botany, Institute of Botany, The Chinese Academy of Sciences, Beijing 100093, China; lanmeifang@ibcas.ac.cn; 3 College of Life Sciences, University of Chinese Academy of Sciences, Beijing 100049, China; 4 School of Life Science, North China University of Science and Technology, Tangshan 063210, China; 5 College of Mathematics and Sciences, North China University of Science and Technology, Tangshan 063210, China

**Keywords:** Aflatoxin B1, isoleucine, high-fat diet, Th17 immune response, gut–liver axis, oxidative stress, intestinal barrier, gut microbiota, inflammation, metabolic dysregulation

## Abstract

**Objectives:** Disruption of gut–liver axis homeostasis is a hallmark of metabolic and toxic stress. This study aimed to evaluate the combined effects of high-fat diet (HFD), aflatoxin B1 (AFB1), and exogenous isoleucine supplementation on immunometabolic function under nutritional and toxic stress. **Methods**: Two-phase murine experiments assessed: (1) HFD and AFB1 effects individually and combined; and (2) dose-dependent isoleucine responses (25/50/100 mg/kg) across control, HFD, and HFD + AFB1 backgrounds. **Results:** HFD significantly impaired liver function, promoted Th17-mediated inflammation, and induced gut dysbiosis, while AFB1 alone exerted minimal effects. Their combination synergistically exacerbated hepatic steatosis, intestinal barrier disruption, and inflammatory responses. Fecal metabolomics identified elevated isoleucine as a potential inflammatory biomarker. Under HFD, isoleucine (50 mg/kg) amplified inflammation and oxidative stress. Remarkably, under HFD + AFB1, moderate/high-dose isoleucine reduced hepatic lipid deposition and triglycerides despite persistent intestinal damage, demonstrating context-dependent effects. **Conclusions:** HFD and AFB1 synergistically disrupt gut–liver axis integrity through immunometabolic mechanisms. Isoleucine supplementation exhibits dual-modulatory effects, exacerbating damage under nutritional stress while partially mitigating hepatic lipid accumulation under combined toxic-nutritional stress, highlighting the critical importance of environmental context in amino acid interventions.

## 1. Introduction

The gut–liver axis is a bidirectional regulatory system that plays a pivotal role in maintaining metabolic homeostasis and immune balance [1,2]. With the rising prevalence of chronic diseases such as non-alcoholic fatty liver disease (NAFLD), type 2 diabetes, and metabolic syndrome, increasing attention has been paid to the influence of exogenous environmental factors on gut–liver dysfunction. Among these, dietary risk factors such as a high-fat diet (HFD) and aflatoxin B1 (AFB1)-commonly co-occurring in modern dietary and food contamination settings-are thought to exert synergistic pathogenic effects [3,4].

HFD is widely used as a model to simulate human metabolic disorders by promoting lipid overload, endotoxin release, and immune activation. Chronic exposure to HFD imposes sustained stress on hepatic metabolism, leading to inflammation, steatosis, mitochondrial dysfunction, and insulin resistance [5,6,7]. Conversely, AFB1, a well-known fungal toxin, can compromise gut barrier integrity and alter microbial composition, subsequently activating hepatic inflammatory pathways [8,9]. The toxic effects of AFB1 are primarily mediated through oxidative stress, DNA damage, and prolonged activation of inflammatory signaling cascades [10]. Although HFD and AFB1 have been independently shown to affect gut and liver function [11], their potential synergistic effects and combined impact on gut–liver axis homeostasis remain insufficiently elucidated.

Recent metabolomic studies have also highlighted the relevance of branched-chain amino acids (BCAAs) in metabolic disease pathophysiology. Isoleucine, a key member of the BCAA family, is not only involved in protein synthesis and energy metabolism but may also modulate immune responses via mTOR and NLRP3 signaling pathways [12,13]. Elevated plasma isoleucine levels have been reported in patients with metabolic syndrome, positively correlating with increased Th17 cell populations, suggesting its role as a potential metabolic trigger of immune dysregulation [14]. However, under stress conditions such as high-fat intake or toxin exposure, the physiological effects of isoleucine may shift toward pathological outcomes. There is currently limited evidence regarding whether exogenous isoleucine contributes to liver and intestinal immune dysfunction under such complex metabolic–toxicological contexts.

To address these questions, this study employed a murine model to comprehensively assess the effects of AFB1 and HFD on gut–liver axis integrity. We further investigated the physiological responses induced by isoleucine supplementation under these conditions. Multiple dimensions were evaluated, including metabolism, inflammation, oxidative stress, and gut microbiota composition. Special emphasis was placed on the Th17 inflammatory axis, redox imbalance, intestinal barrier protein expression, and microbial ecology. This study aimed to provide fundamental data for understanding how functional amino acids modulate gut–liver homeostasis under dual nutritional and toxicological stress.

## 2. Materials and Methods

### 2.1. Experimental Animals and Housing Conditions

Specific pathogen-free (SPF) male C57BL/6J mice aged 6 weeks (body weight: 20–24 g) were obtained from Huafukang Laboratory Animal Technology Co., Ltd. (Beijing, China; License No. SCXK (Jing) 2024-0003). All animal experiments were conducted at the animal facility of Tianjin Guosheng Zhongyuan Technology Co., Ltd. (Tianjin, China; License No. SYKZ (Jin) 2021-0003). Mice were housed under standard laboratory conditions with a controlled temperature of 22 ± 2 °C, relative humidity of 50–60%, and a 12 h light/dark cycle. All animals had ad libitum access to food and water. A 7-day acclimatization period was allowed prior to the experimental interventions.

### 2.2. Diet and Reagents

Standard chow was provided by Beijing Vital River Laboratory Animal Technology Co., Ltd. (Beijing, China). The high-fat diet (HFD) was formulated to derive 60% of energy from fat, 20% from carbohydrates, and 20% from protein (based on D12492), to induce metabolic disturbance. Aflatoxin B1 (AFB1) was purchased from Shanghai Yuanye Bio-Technology Co., Ltd. (Shanghai, China), dissolved in 0.5% dimethyl sulfoxide (DMSO), and diluted with sterile saline to working concentration. Isoleucine was obtained from Sigma-Aldrich (St. Louis, MO, USA) and freshly dissolved in sterile saline prior to administration.

### 2.3. Experimental Design and Treatment Protocol

The study consisted of two phases:

Phase I (AFB1 and HFD Modeling): A total of 32 mice were randomly divided into four groups (n = 8 per group): Ctrl (standard chow), AFB1 (standard chow + AFB1 by gavage), HFD (high-fat diet), and AFB1 + HFD (HFD + AFB1 by gavage). The first two weeks involved diet adaptation. From week 3 onward, HFD and AFB1 + HFD groups were fed a high-fat diet, and mice in the AFB1 and AFB1 + HFD groups received daily gavage of AFB1 (200 μg/kg·bw) until week 18. Body weight was recorded weekly. At the end of the experiment, liver, ileum, and epididymal fat were collected for analysis.

Phase II (Isoleucine Intervention): A total of 72 mice were randomly assigned to 9 groups (n = 8 per group): Ctrl + Ile25/50/100 (standard chow + 25, 50, or 100 mg/kg·bw isoleucine), HFD + Ile25/50/100, and HFD + AFB1 + Ile25/50/100. During weeks 0–2, mice underwent diet adaptation and modeling. From week 3, HFD groups received high-fat diet, and AFB1 groups received daily gavage of AFB1 (200 µg/kg·bw) through week 18. In week 19, mice were intraperitoneally injected with the assigned dose of isoleucine once daily for 5 consecutive days. At the end of the study, body weight was recorded and blood, liver, ileum, and adipose tissues were collected for analysis.

### 2.4. Hematoxylin and Eosin (H&E) Staining

Liver and ileum tissues were fixed in 10% neutral buffered formalin (pH 7.4, Solarbio Life Sciences, Beijing, China) for 24 h, dehydrated, paraffin-embedded, and sectioned at 4 μm. Sections were deparaffinized with xylene and rehydrated through graded ethanol. Nuclei were stained with hematoxylin for 3 min, differentiated with 0.5% acid ethanol for 15 s, blued in alkaline ethanol, and counterstained with eosin for 90 s. After dehydration and mounting, images were acquired using an Olympus BX53 microscope (Olympus Corporation, Tokyo, Japan), and analyzed with ImageJ 1.52a (NIH, Bethseda, MD, USA).

### 2.5. Oil Red O Staining

Liver tissues were rinsed with cold PBS and sectioned at 8 μm using OCT-embedded frozen blocks stored at −20 °C. Sections were stained with freshly prepared 0.5% Oil Red O solution (Sigma-Aldrich, USA) for 15 min, differentiated with 75% ethanol, and counterstained with DAPI for 5 min (Beyotime Biotechnology, Shanghai, China). Lipid droplets were imaged using a Leica DMi8 fluorescence microscope (Leica Microsystems, Wetzlar, Germany) and quantified using ImageJ.

### 2.6. Immunofluorescence Staining

Frozen ileal sections (6–8 μm) were washed with PBS, permeabilized with 0.3% Triton X-100 for 10 min, and blocked with 5% BSA for 1 h. Primary antibodies—rabbit anti-IL-17A (1:200, Abcam, Cambridge, UK) and mouse anti-CD4 (1:200, Proteintech, Rosemont, IL, USA)—were applied overnight at 4 °C. Sections were incubated with Alexa Fluor 594-conjugated goat anti-rabbit IgG and Alexa Fluor 488-conjugated goat anti-mouse IgG (1:500, Thermo Fisher Scientific, Waltham, MA, USA) for 1 h at 37 °C, followed by DAPI nuclear staining. Imaging was conducted on an Olympus FV3000 confocal microscope (Olympus, Japan).

### 2.7. Serum Biochemical Assays

Blood samples collected from the abdominal aorta were centrifuged at 3000 rpm for 10 min at 4 °C to separate serum. Alanine aminotransferase (ALT), aspartate aminotransferase (AST), albumin (ALB), total cholesterol (TC), and triglycerides (TG) were measured using commercial kits (Nanjing Jiancheng Bioengineering Institute, Nanjing, China) according to the manufacturer’s instructions. Absorbance was read at 450 nm using a BioTek Synergy HTX microplate reader (BioTek Instruments, Winooski, VT, USA), with all samples analyzed in triplicate.

### 2.8. Oxidative Stress Biomarkers

Liver homogenates were used to assess reduced glutathione (GSH), superoxide dismutase (SOD), and malondialdehyde (MDA) levels using commercial assay kits (Nanjing Jiancheng Bioengineering Institute, Nanjing, China). GSH was determined using the DTNB method, SOD by the xanthine oxidase method, and MDA by the TBA method. Absorbance was measured at 412, 550, and 532 nm, respectively. All results were normalized to protein content and expressed as μmol/g protein or U/mg protein.

### 2.9. ELISA for Cytokine Quantification

Concentrations of IL-6, IL-22, and TGF-β in serum were determined using ELISA kits (Multi Sciences, Hangzhou, China), following the manufacturer’s protocols, and data were expressed as pg/mL.

### 2.10. Mononuclear Cell Isolation and Flow Cytometry Analysis

Mononuclear cells were isolated from liver and peripheral blood by density gradient centrifugation. Fc receptors were blocked to minimize non-specific binding. Cells were stained with FITC-conjugated anti-CD4 and PE-conjugated anti-IL-17A antibodies (BioLegend, San Diego, CA, USA) for 30 min at 4 °C in the dark. After washing with PBS, samples were analyzed on a BD FACSCanto II flow cytometer (BD Biosciences, Franklin Lakes, NJ, USA), and data were processed using FlowJo v10.6.2 (BD Biosciences, USA).

Liver non-parenchymal cell isolation: Mice underwent PBS portal vein perfusion followed by mechanical and enzymatic dissociation using Miltenyi Liver Dissociation Kit—Miltenyi Biotec, Auburn, CA, USA (37 °C, program 37C_m_LIDK_1). The cell suspension was filtered through a 100 μm cell strainer, centrifuged at 300× *g* for 10 min, resuspended in pre-chilled PBS with debris removal solution, and centrifuged at 3000× *g* for 10 min with reduced acceleration rate (level 4/9) to ensure well-defined phase separation. For red blood cell contamination, ACK lysis buffer was applied on ice for 2 min followed by PBS wash and centrifugation at 500× *g* for 5 min. Peripheral blood mononuclear cell isolation: PBMCs were isolated by Ficoll density gradient centrifugation at 500× *g* for 30 min, with interface layer cells collected.

Cell stimulation and staining: Cells were stimulated with PMA (50 ng/mL) and ionomycin (1 μg/mL) for 4 h with Brefeldin A (10 μg/mL) blockade. Fixation and permeabilization were performed using eBioscience^TM^ Fixation & Permeabilization Buffer Kit (Thermo Fisher Scientific, Waltham, MA, USA). Surface staining was conducted with FITC Anti-CD4 (clone RM4-5, abcam) and PE Anti-IL-17A (clone eBioTB-7, Lianke Biotechnology, Hangzhou, China) for 30 min at 4 °C in the dark. All samples included FMO controls.

Flow cytometry analysis: Data were acquired on BD FACSCanto II flow cytometer and analyzed using FlowJo v10.6.2. Gating strategy: (1) FSC-A/FSC-H for doublet exclusion; (2) DAPI for dead cell exclusion; (3) CD45^+^ leukocyte gate; (4) CD3^+^ T cell gate; (5) CD4^+^ helper T cell gate; (6) IL-17A^+^ Th17 cell identification. Th17 cells were defined as CD3^+^ CD4^+^ IL-17A^+^, expressed as percentage of CD4^+^ T cells.

### 2.11. 16S rRNA Sequencing and Fecal Metabolomics Analysis

Microbial DNA was extracted from ileal contents using the TIANGEN Fecal DNA Kit (TIANGEN Biotech, Beijing, China). The V3–V4 region of the 16S rRNA gene was amplified and sequenced using the Illumina NovaSeq platform by Shanghai Weipu Biotechnology Co., Ltd. (Shanghai, China). Raw data were processed using QIIME2 (version 2020.11) to analyze α-diversity and β-diversity (principal coordinates analysis (PCoA)). Species annotation and plotting were conducted using R 4.0 software. To identify differentially abundant taxa, Linear Discriminant Analysis Effect Size (LEfSe) analysis was applied with an LDA threshold of >2.0, and statistical significance was determined using a *p*-value threshold of <0.05 after multiple testing corrections. Additionally, a random forest model was used to assess the importance of bacterial genera, with the Mean Decrease Gini coefficient as the criterion for evaluation. Each treatment group consisted of 5 mice (n = 5), including Ctrl, AFB1, HFD, and AFB1 + HFD.

Untargeted fecal metabolomics was performed using a Vanquish UPLC system (Thermo Fisher Scientific, Waltham, MA, USA) coupled to a Q-Exactive HFX Orbitrap mass spectrometer (Thermo Fisher Scientific, Waltham, MA, USA). Chromatographic separation was achieved using an HSS T3 column (100 × 2.1 mm, 1.8 μm; Waters Corporation, Milford, MA, USA) at 40 °C. The flow rate was 0.3 mL/min and the injection volume was 2 μL. The mobile phases were Milli-Q water with 0.1% formic acid (A) and acetonitrile with 0.1% formic acid (B), using a linear gradient. Data acquisition was conducted with Xcalibur 4.1 software (Thermo Fisher Scientific, Waltham, MA, USA), followed by preprocessing using Progenesis QI (Waters Corporation, Milford, MA, USA). Multivariate statistical analyses were performed using SIMCA-P software (version 14.1; Sartorius Stedim Data Analytics AB, Umeå, Sweden).

### 2.12. Isolation of Primary CD4^+^ T Cells and Isoleucine Treatment

Mice were sacrificed under sterile conditions, and the livers were perfused to remove residual blood. Liver tissue was minced and mechanically homogenized to obtain a single-cell suspension. After filtering through a 70 μm filter, liver-infiltrating mononuclear cells (LIMCs) were obtained by centrifugation on a Percoll density gradient (40%/70%) (800× *g*, 20 min, room temperature). Peripheral blood was obtained by retroorbital/cardiac puncture, anticoagulated with EDTA, and PBMCs were obtained by Ficoll-Paque density gradient centrifugation (400× *g*, 30 min, no brake). Single-cell suspensions were prepared from mouse spleens (70 μm filter) and lysed using Acute Cytotoxicity (ACK) to remove erythrocytes. After adding BD Fc Block^TM^ (0.25 μg/10^6^ cells, 15 min, 4 °C), cells were incubated with anti-mouse CD4 magnetic beads (BD Biosciences) according to the manufacturer’s instructions (50 μL/10^7^ cells, 30 min, 4 °C). CD4^+^ T cells were isolated by magnetic column sorting. Flow cytometry reassessment revealed a purity of >90%.

Cells were cultured in RPMI-1640 medium with 10% FBS, 1% penicillin-streptomycin, 1 mmol/L sodium pyruvate, 1 mmol/L glutamine, 1 mmol/L β-mercaptoethanol, and 10 ng/mL each of IL-2 and IL-7. After seeding at 1×10^6^ cells/mL in 6-well plates, cells were treated immediately with 1 mmol/L DMSO or isoleucine (dissolved in DMSO) for 72 h. For the last 4 h of the 72 h culture, cells were stimulated with PMA (50 ng/mL), ionomycin (1 μg/mL), and Brefeldin A (10 μg/mL).

Fixable Viability Dye ensures that live/dead exclusion remains stable after fixation/permeabilization. For flow cytometric analysis, cells were fixed/permeabilized using eBioscience^TM^ Fixation & Permeabilization Kit, stained with APC-conjugated anti-CD45 (clone 30-F11, BioLegend, USA), PerCP-Cy5.5-conjugated anti-CD3 (clone 17A2, BioLegend, USA), FITC-anti-CD4 (clone RM4-5, Abcam) and PE-anti-IL-17A (clone eBioTB-7, Lianke Biotechnology) for 30 min at 4 °C in the dark. FMO and unstimulated controls were included for proper compensation and gating. After washing, samples were analyzed using a BD FACSCalibur flow cytometer and data processed with FlowJo v10.6.2. Gating strategy: (1) FSC-A/SSC for lymphocyte identification; (2) FSC-A/FSC-H for doublet exclusion; (3) DAPI for dead cell exclusion; (4) CD45^+^ leukocyte gate; (5) CD3^+^ T cell gate; (6) CD4^+^ T cell gate; (7) IL-17A^+^ Th17 cell identification. Th17 cells were defined as CD3^+^CD4^+^IL-17A^+^, expressed as a percentage of CD4^+^ T cells. All gating decisions were based on FMO controls and unstimulated samples for IL-17A specificity.

### 2.13. Statistical Analysis

All data are presented as mean ± standard deviation (SD). Statistical significance between groups was assessed by one-way analysis of variance (ANOVA) followed by Tukey’s post hoc test using GraphPad Prism 10.0 (GraphPad Software, Boston, MA, USA). A *p*-value of * *p* < 0.05, ** *p* < 0.01, *** *p* < 0.001, and **** *p* < 0.0001 was considered statistically significant.

## 3. Results

### 3.1. Effects of AFB1, HFD, and Their Combination on Body Weigh, Organ Indices, Liver Function and Lipid Metabolism in Mice

**Figure 1:**
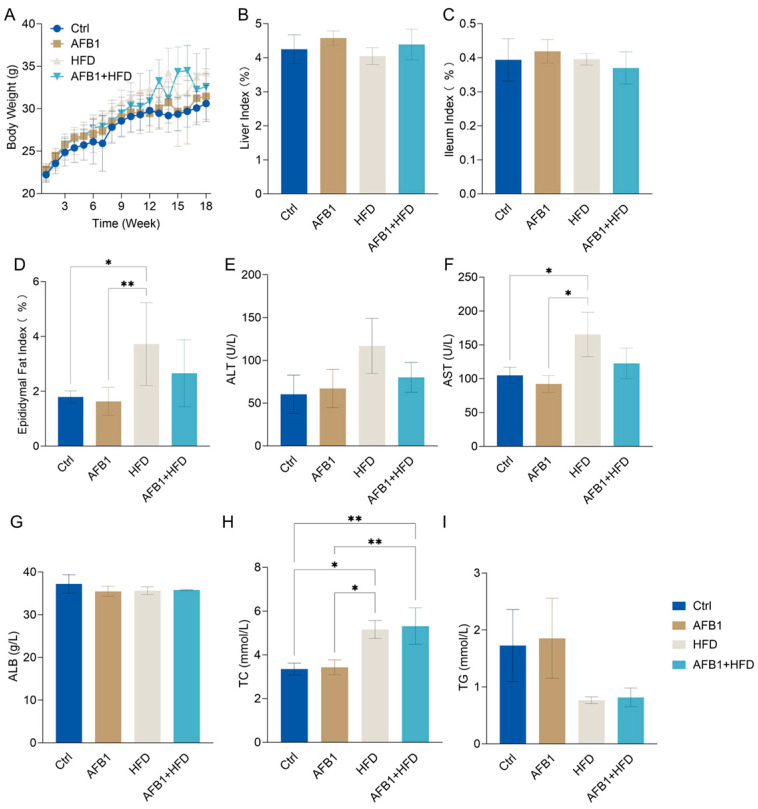
Effects of AFB1, HFD, and their combined intervention on body weight progression, metabolic organ indices, and serum biochemical parameters in mice (n = 8, mean ± SEM). (**A**) Body weight trajectory over the 18-week intervention period; (**B**) Liver index (%); (**C**) Ileum index (%); (**D**) Epididymal fat index (%); (**E**) ALT, U/L; (**F**) AST, U/L; (**G**) ALB, g/L; (**H**) TC, mmol/L; (**I**) TG, mmol/L. * *p* < 0.05, ** *p* < 0.01.

To elucidate the impact of AFB1, HFD, and their co-exposure on mice, a longitudinal assessment was performed over an 18-week period. As illustrated in Figure 1A, HFD-fed mice exhibited a significant increase in body weight beginning at week 6 compared to the control group (Ctrl, *p* < 0.01), reaching a maximum of 34.28 ± 1.15  g by week 18, the highest among all groups. The AFB1-only group reached 31.45 ± 1.10  g, slightly higher than the Ctrl group (30.60 ± 0.87  g, *p* > 0.05), suggesting that low-dose AFB1 alone had a minimal impact on body weight under standard dietary conditions. The combined AFB1 + HFD group showed a notable deviation in weight gain from the Ctrl group starting at week 9, with a final weight of 32.59 ± 0.86  g (*p* < 0.05) at week 18.

Regarding organ indices, no significant differences in liver weight relative to body weight were observed across groups (Figure 1B; *p* > 0.05). The liver index was 4.25 ± 0.17% in the Ctrl group, marginally increased in the AFB1 group (4.58 ± 0.09%), and slightly reduced in the HFD group (4.05 ± 0.10%), indicating no marked hepatic hypertrophy or atrophy after 18 weeks of intervention. Similarly, the ileum index remained comparable across groups (Figure 1C; *p* > 0.05), with a modest increase in the AFB1 group (0.419 ± 0.014%) compared to the Ctrl (0.394 ± 0.025%), suggesting potential mild intestinal structural stress. Regarding the epididymal fat index, a significant increase was observed in the HFD group (3.72 ± 0.62%, *p* < 0.05). Although the AFB1 + HFD group also showed an upward trend (2.66 ± 0.50%), this did not reach statistical significance (*p* > 0.05). No significant difference was found between the AFB1-only group (1.63 ± 0.21%) and the control group (1.79 ± 0.09%) (Figure 1D).

Figure 1E showed elevated ALT in the HFD group (120.13 ± 44.53 U/L), though not significantly different from Ctrl or AFB1 groups. Conversely, HFD group AST (Figure 1F, 165.37 ± 32.76 U/L) was significantly higher than both Ctrl and AFB1 (*p* < 0.05). AFB1 alone had minimal impact on liver enzymes in ALT, AST or ALB (Figure 1E–G). In the AFB1 + HFD group, ALT and AST levels (80.13 ± 17.46 U/L and 122.67 ± 22.56 U/L) were slightly higher than in the Ctrl group, but still not significant. Notably, ALT levels in this group were even lower than in the HFD group. ALB levels remained stable across all groups (35–37 g/L, *p* > 0.05), suggesting relieved hepatic protein synthesis capacity.

Regarding lipid metabolism as shown in Figure 1H–I, TC levels were markedly elevated in the HFD group (5.16 ± 0.41 mmol/L, *p* < 0.05 vs. Ctrl). Elevated TC concentrations were also observed in the AFB1 + HFD group (5.31 ± 0.83 mmol/L, *p* < 0.05 vs. Ctrl), suggesting that such intervention disrupted cholesterol homeostasis. Although TG concentrations were lower in all treatment groups compared to the Ctrl (1.73 ± 0.63 mmol/L), the differences did not reach statistical significance. TG levels in both the HFD and AFB1 + HFD groups remained at relatively low levels (0.77 ± 0.06 and 0.82 ± 0.17 mmol/L, respectively). AFB1 alone had no apparent effect on serum TG.

In summary, HFD primarily increased hepatic enzyme levels and cholesterol accumulation, while AFB1 alone had minimal effect on liver function or lipid metabolism. Combined AFB1 + HFD exposure did not exhibit synergistic toxicity and even slightly reduced ALT and AST, indicating a potential adaptive response.

### 3.2. Histological and Immunofluorescence Analysis of Ileal and Hepatic Alterations

**Figure 2:**
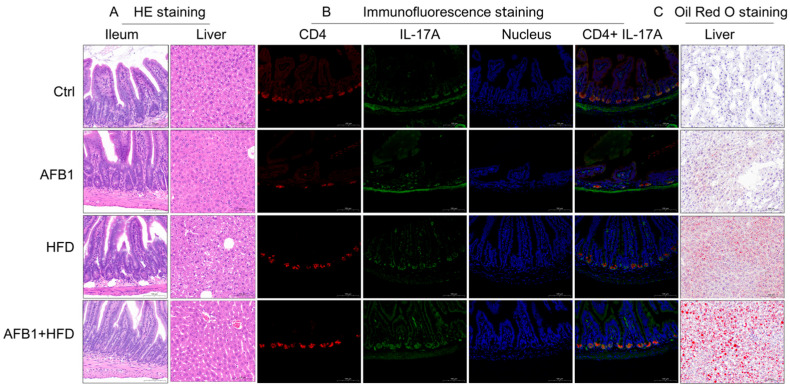
Histological, immunofluorescent, and lipid deposition changes in the ileum or/and liver of mice exposed to AFB1, HFD, or their combination. (**A**) H&E staining of ileal and hepatic tissues (ileum, scale bar = 100 μm; liver, scale bar = 50 μm); (**B**) Immunofluorescence analysis of CD4^+^ and IL-17A^+^ co-localization in ileal tissues; scale bar = 100 μm; (**C**) Oil Red O staining of hepatic lipid droplets, scale bar = 100 μm.

To explore tissue-level alterations induced by AFB1, HFD, and their combined exposure, histological, immunofluorescent, and lipid staining analyses were conducted as shown in Figure 2.

H&E staining showed distinct ileal and hepatic pathological alterations among treatment groups. The Ctrl group had intact ileal morphology with well-organized villi, crypts, and mucosa, while the AFB1 group maintained an intact ileal mucosal structure with orderly crypts and no obvious inflammation. The HFD group showed increased crypt depth with active cell proliferation at the crypt base and inflammatory cell infiltration. Notably, the AFB1 + HFD group presented more severe ileal damage, with increased crypt depth, active cell proliferation at the crypt base, inflammatory cell infiltration, reduced number of goblet cells, and decreased intracellular mucus vacuoles.

In the liver, Ctrl group hepatocytes showed orderly hepatic cords with no steatosis or necrosis. The AFB1 group maintained overall structure with mild cytoplasmic vacuolization. The HFD group exhibited microvesicular steatosis with variably sized, well-demarcated circular vacuoles in the cytoplasm. The AFB1 + HFD group showed microvesicular steatosis with variably sized, well-demarcated circular vacuoles, with some cell nuclei displaced to the periphery, indicating enhanced metabolic stress under co-exposure.

Immunofluorescence staining clarified Th17 pathway activation. Ctrl ileum exhibited low CD4 and IL-17A expression with minimal co-localization. HFD increased CD4^+^ and IL-17A^+^ cell counts and fluorescence intensity. AFB1 alone had negligible effect, mirroring Ctrl levels. Strikingly, the AFB1 + HFD group demonstrated a pronounced rise in both markers, with expanded co-localized regions and stronger fluorescence, supporting Th17 activation under dual insult.

Oil Red O staining demonstrated hepatic lipid accumulation patterns. Ctrl livers lacked lipid droplets and retained structural integrity. The HFD group developed small, densely packed droplets, while the AFB1 group had only sparse deposits. The AFB1 + HFD group showed significantly larger, clustered droplets, some displacing hepatocyte nuclei—hallmarks of aggravated steatosis and hepatocellular injury.

Together, these observations suggest that AFB1 or HFD alone induce limited pathology, whereas their combination leads to pronounced intestinal barrier disruption, hepatic lipid accumulation, and Th17-mediated inflammation. It is important to note that this synergistic effect is observed across multiple pathological endpoints but not in body weight gain, where AFB1 appears to partially counteract HFD-induced weight increase. This distinction highlights that AFB1 and HFD interact differently with various physiological systems.

### 3.3. Combined Effects of AFB1 and HFD on Inflammation, Oxidative Stress, and Intestinal Barrier Integrity

**Figure 3:**
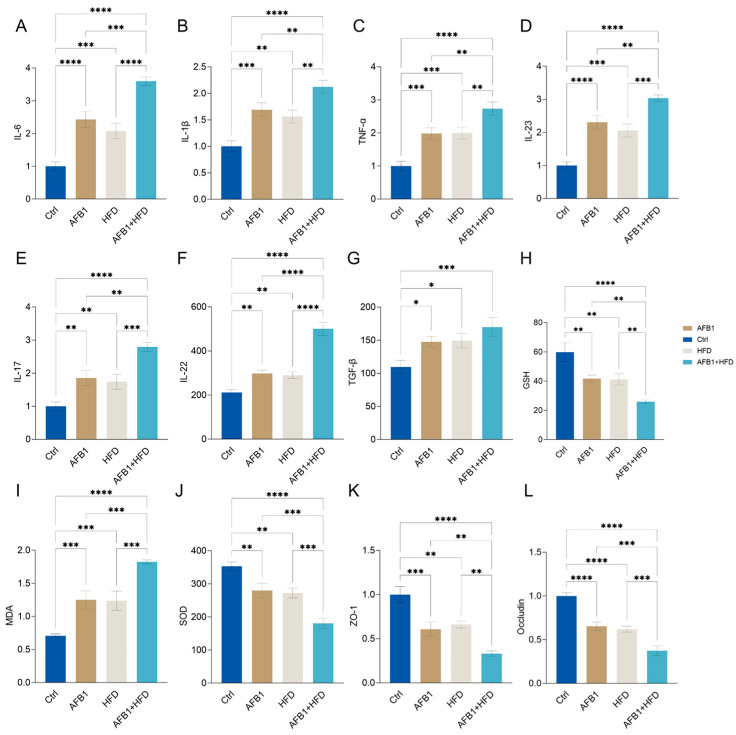
Effects of AFB1, HFD, and their combination on serum proinflammatory cytokines, oxidative stress markers, and intestinal tight junction protein expression in mice (n = 3, mean ± SD). (**A**) IL-6; (**B**) IL-1β; (**C**) TNF-α; (**D**) IL-23; (**E**) IL-17; (**F**) IL-22; (**G**) TGF-β; (**H**) MDA (nmol/mg); (**I**) GSH (μmol/g); (**J**) SOD (U/mg); (**K**) ZO-1 (relative expression); (**L**) Occludin (relative expression). * *p* < 0.05, ** *p* < 0.01, *** *p* < 0.001, and **** *p* < 0.0001.

The effects of AFB1 and HFD on inflammatory responses, oxidative stress, and intestinal barrier homeostasis, twelve key biological indicators were analyzed, including serum cytokines, redox markers, and tight junction proteins as shown in Figure 3.

Both AFB1 and HFD alone significantly increased serum levels of cytokines including IL-6, IL-1β, TNF-α, IL-23, IL-17, IL-22, and TGF-β. Notably, these elevating effects were further amplified in the combined treatment group compared to the HFD or AFB1 alone groups (*p* < 0.05 or lower), with the exception of TGF-β, indicating a pronounced synergistic proinflammatory response.

Regarding oxidative stress, the serum concentrations of MDA were significantly elevated in the combined group (*p* < 0.0001), while the levels of endogenous antioxidants, GSH and SOD, were significantly reduced (both *p* < 0.0001), reflecting impaired antioxidant capacity and increased oxidative stress. Furthermore, the expression levels of tight junction proteins ZO-1 and Occludin were significantly lower in the combined group (both *p* < 0.0001), suggesting compromised intestinal barrier integrity. This structural disruption may facilitate endotoxin translocation, thereby exacerbating hepatic inflammatory damage. Notably, the combined group exhibited synergistic effects, with MDA levels significantly higher (*p* < 0.05 or lower) than those in the individual treatment groups, and GSH and SOD levels significantly lower (*p* < 0.05 or lower), further supporting the enhanced oxidative burden and barrier dysfunction observed in this group.

In summary, AFB1 and HFD produce a pronounced synergistic disruption of gut–liver axis homeostasis via a three-pronged mechanism—heightened proinflammatory signaling, amplified oxidative stress, and marked impairment of the intestinal epithelial barrier.

### 3.4. Combined Effects of AFB1 and HFD on the Th17 Immune Axis in Liver and Peripheral Blood

**Figure 4:**
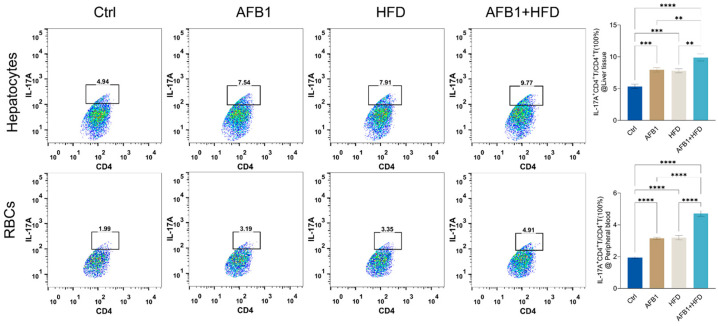
Proportional changes in CD4^+^IL-17A^+^ Th17 cells in hepatic and peripheral blood compartments of mice following exposure to AFB1, HFD, or their combination, as assessed by flow cytometry. Upper panels represent hepatocyte-derived lymphocytes; lower panels represent PBMCs. Statistical summary of the IL-17A^+^CD4^+^T/CD4^+^T proportions were from three independent experiments (n = 3, mean ± SEM). Gates indicate CD4^+^IL-17A^+^ populations, with percentages reflecting the proportion of Th17 cells within the CD4^+^ T-cell subset. ** *p* < 0.01, *** *p* < 0.001, and **** *p* < 0.0001.

This study employed flow cytometry to evaluate the effects of AFB1, HFD, and their combined intervention on the Th17 immune axis in mice by quantifying CD4^+^IL-17A^+^ T cells (Th17 cells) as a proportion of total CD4^+^ T cells in both liver tissue and peripheral blood in Figure 4.

In hepatic samples, the proportion of Th17 cells progressively increased from the control group (5.34 ± 0.34%) to the AFB1 group (7.95 ± 0.36%) and the HFD group (7.80 ± 0.32%), reaching the highest level in the AFB1 + HFD co-treatment group (9.89 ± 0.56%). These results indicate that the combined intervention significantly enhanced Th17-mediated hepatic inflammation. A similar trend was observed in peripheral blood, where Th17 frequencies rose from 1.94 ± 0.06% in the control group to 3.15 ± 0.06% and 3.20 ± 0.14% in the AFB1 and HFD groups, respectively, and further to 4.71 ± 0.18% in the combined group, reflecting a pronounced systemic activation of the Th17 response. These findings demonstrate that both AFB1 and HFD independently promote Th17 cell expansion, while their co-administration exerts a synergistic effect, particularly evident in hepatic tissue.

### 3.5. Impact of AFB1 and HFD on Gut Microbiota Diversity and Composition

**Figure 5:**
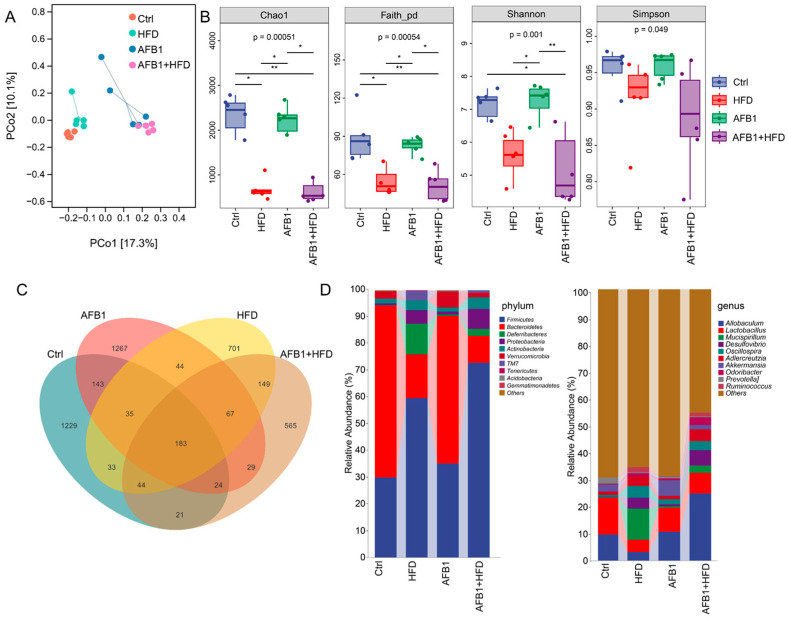
Effects of AFB1, HFD, and their combination on gut microbiota diversity and composition in mice (n = 5, mean ± SD). (**A**) PCoA based on Bray–Curtis distance; (**B**) α-diversity indices (Chao1, Faith’s phylogenetic diversity [Faith_pd], Shannon, and Simpson); (**C**) Venn diagram on OTUs; (**D**) Taxonomic distribution at the phylum level and the genus level. * *p* < 0.05, ** *p* < 0.01.

To comprehensively assess the impact of different exposures on gut microbial homeostasis, we performed multilevel analyses including β-diversity, α-diversity, operational taxonomic unit (OTU) characteristics, and community composition. PCoA (Figure 5A) revealed distinct clustering patterns across groups. The Ctrl group exhibited tight clustering, whereas the AFB1 and HFD groups diverged along separate axes. Notably, the AFB1 + HFD group formed an independent cluster, indicating that the dual intervention induced a novel and distinct microbial state.

As shown in Figure 5B, significant differences in microbial α-diversity were observed among treatment groups. The Chao1 and Faith_pd indices indicated that the HFD group had significantly lower microbial richness than the Ctrl and AFB1 groups (*p* < 0.05), with the AFB1 + HFD group showing the same trend. The Shannon index revealed a slight increase in the AFB1 group compared to the Ctrl group, but a significant decrease in the HFD group (*p* < 0.05 vs. AFB1), and the lowest value in the AFB1 + HFD group (*p* < 0.01 vs. AFB1). The Simpson index showed no significant differences. In summary, HFD primarily reduced microbial diversity, and its combination with AFB1 had an amplified inhibitory effect.

Venn diagram analysis of OTUs (Figure 5C) identified 183 core OTUs shared across all groups. The AFB1group had the highest number of unique OTUs (1267), followed by the Ctrl group (1229), while the AFB1 + HFD group exhibited the fewest (565), indicating that the dual intervention imposed the strongest selective pressure on rare taxa.

At the phylum level (Figure 5D), the AFB1 + HFD group showed a marked shift in microbial composition, characterized by a substantial increase in Firmicutes and a decline in Bacteroidetes, reversing the typical F/B ratio. Proinflammatory phyla such as Proteobacteria and Actinobacteria were also elevated compared to the Ctrl group. Notably, the microbial profiles of the HFD and AFB1 + HFD groups were highly similar, as were those of the Ctrl and AFB1 groups, indicating that AFB1 alone had a limited effect, while HFD was the primary driver of phylum-level alterations. At the genus level, AFB1 treatment enriched certain beneficial taxa like *Allobaculum* and *Akkermansia*, whereas HFD promoted the growth of potentially harmful genera such as *Desulfovibrio* and *Mucispirillum*. In the combined group, *Allobaculum* increased further, while *Akkermansia* markedly declined, suggesting a synergistic depletion of barrier-protective microbes under dual exposure.

These findings indicate that AFB1 and HFD disrupt the intestinal microbiota via distinct but complementary mechanisms-AFB1 suppresses dominant taxa while transiently enriching rare species, whereas HFD simplifies microbial structure and enriches pathobionts.

### 3.6. Impact of AFB1 and HFD on Gut Microbiota Characteristics and Enrichment of Proinflammatory Taxa

**Figure 6:**
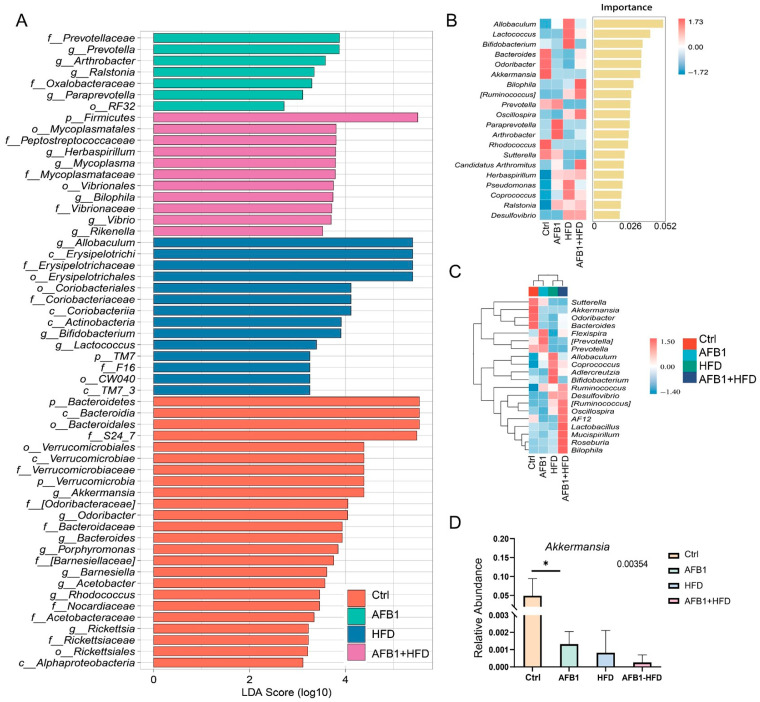
Effects of AFB1, HFD, and their combination on gut microbiota composition and key genera in mice. (**A**) LEfSe analysis identifying bacterial taxa significantly enriched in each group (LDA > 2.0); (**B**) Random forest model ranking genera based on their importance in group differentiation; (**C**) Heatmap clustering of microbial community composition across treatment groups; (**D**) Relative abundance of *Akkermansia* (n = 5, mean ± SEM). * *p* < 0.05.

Figure 6 provides a comprehensive overview of the gut microbiota alterations induced by AFB1, HFD, and their combined treatment in mice. In the Ctrl group, dominant taxa included the phylum Bacteroidetes, the genus *Akkermansia* from the Verrucomicrobia phylum, and fiber-degrading and SCFA-producing genera such as *Odoribacter*, *Barnesiella*, and *Porphyromonas*. These profiles indicate a functionally diverse and metabolically balanced microbial community associated with mucosal homeostasis and anti-inflammatory status. In the AFB1 group, the microbiota exhibited moderate restructuring characterized by the enrichment of *Prevotella*, *Paraprevotella*, *Arthrobacter*, and *Ralstonia*, alongside taxa from *Prevotellaceae* and *Oxalobacteraceae*. These shifts predominantly involved stress-responsive and rare taxa, suggesting that low-dose AFB1 alone exerted a limited microbiota-disrupting effect, potentially inducing adaptive metabolic remodeling. The HFD group displayed profound alterations in microbiota composition, marked by the enrichment of lipid metabolism-associated genera such as *Allobaculum*, *Erysipelotrichaceae*, and *Coriobacteriaceae* [15]. The AFB1 + HFD group showed the most pronounced dysbiosis, with strong enrichment of *Firmicutes*, *Mycoplasmataceae*, *Peptostreptococcaceae*, *Bilophila*, *Rikenella*, and *Vibrio*. These taxa are closely linked to mucosal damage, LPS production, and TLR4 signaling activation [16,17,18], indicating a proinflammatory, barrier-compromising microbial environment.

Random forest analysis (Figure 6B) identified *Allobaculum* as the most important genus for group differentiation (importance score = 0.052), followed by *Lactococcus* and *Bifidobacterium*. Notably, *Akkermansia* exhibited the highest relative abundance in the Ctrl group and decreased across the AFB1, HFD, and AFB1 + HFD groups. Heatmap clustering (Figure 6C) further revealed pronounced compositional divergence in the AFB1 + HFD group, which was enriched in proinflammatory genera such as *Ruminococcus*, *Bilophila*, and *Oscillospira*. These genera sharply contrasted with the *Akkermansia*-dominant profile in the Ctrl group, highlighting a shift from a protective to a pathogenic microbial landscape under combined nutritional and toxic stress.

Quantitative analysis of the key symbiotic bacterium *Akkermansia* (Figure 6D) showed the highest relative abundance in the Ctrl group, while its levels significantly declined in the AFB1, HFD, and combined groups. The reduction was most pronounced in the AFB1 + HFD group. Given that *Akkermansia* plays a critical role in maintaining intestinal barrier integrity and regulating mucosal immunity, its depletion suggests that both toxin exposure and dietary fat may compromise epithelial defenses and promote inflammatory signaling.

Overall, AFB1 and HFD exerted a synergistic proinflammatory effect on gut microbiota structure, characterized by the expansion of pathobionts and depletion of beneficial microbes.

### 3.7. Metabolomic Analysis of Fecal Samples and Isoleucine Supplementation on CD4 ^+^ T Cells

Amino acids are critical biomarkers of inflammation and play a key role in metabolic disorders. These amino acids are recognized as important markers of inflammation, reflecting the presence of metabolic inflammation [19].

**Figure 7:**
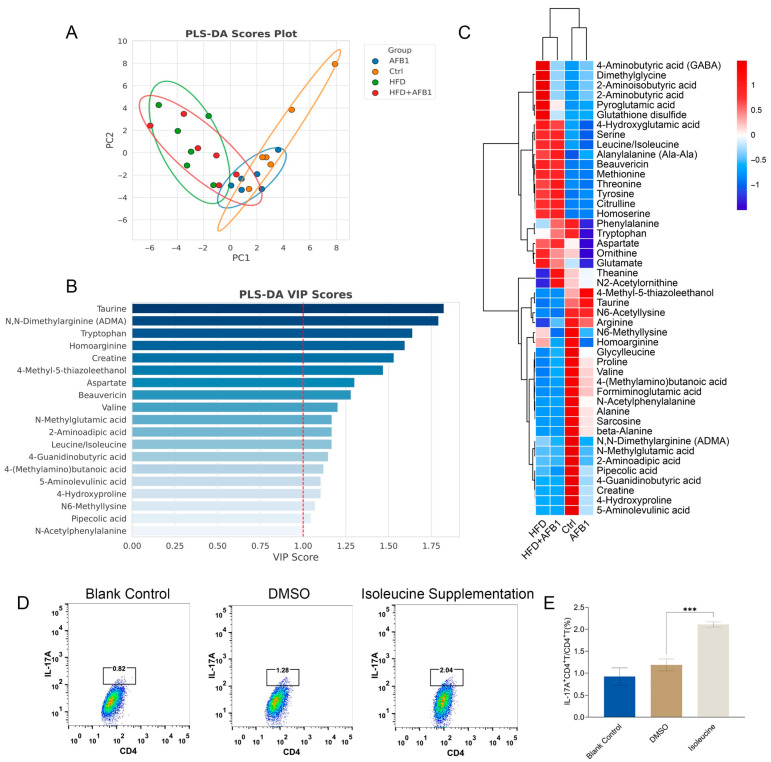
Fecal metabolomic profiles and CD4^+^ T cell responses following isoleucine supplementation. (**A**) PLS-DA score plot; (**B**) VIP plot; (**C**) Heatmap of differentially abundant amino acids across treatment groups; (**D**) Representative flow cytometry plots showing CD4^+^ T cell populations in blank control, DMSO, and isoleucine supplementation groups; (**E**) Statistical summary of CD4^+^ T cell proportions from three independent experiments (n = 3, mean ± SEM). *** *p* < 0.001.

To explore the role of these amino acids, we performed metabolomic analysis on fecal samples, focusing on the abundance of amino acids. PLS-DA analysis (Figure 7A) showed that the HFD and HFD + AFB1 groups exhibited high similarity. Similarly, the AFB1 and Ctrl groups overlapped considerably. Notably, significant separation was observed between the two major groups: AFB1 vs. Ctrl and HFD vs. HFD + AFB1, indicating distinct effects of the treatments on amino acid metabolism.

In the VIP plot (Figure 7B), isoleucine/leucine (the mass spectrum did not distinguish between them) was identified as a key variable with VIP > 1 and was significantly elevated in both the HFD and HFD + AFB1 groups, as shown in the heatmap (Figure 7C, *p* <0.001), suggesting metabolic changes under these conditions. Several studies have shown that branched-chain amino acids (BCAAs), including leucine and isoleucine, are elevated in systemic inflammation and are positively correlated with inflammatory markers such as IL-6 and TNF-α [20,21]. Compared to other BCAAs, isoleucine has a stronger inflammatory signature closely associated with the activation of the NLRP3 inflammasome [22,23]. Therefore, the elevated levels of isoleucine in the HFD and HFD + AFB1 groups may indicate its potential as an inflammatory biomarker under these conditions.

Furthermore, to validate the effects of isoleucine, we assessed its impact on immune modulation and inflammation pathways. Isoleucine supplementation significantly increased the ratio of IL-17A ^+^ CD4 ^+^ T cells/CD4 ^+^ T cells, as shown in the flow cytometry data (Figure 7D). This increase was particularly evident in the isoleucine supplementation group, where the IL-17A ^+^ CD4 ^+^ T cell ratio was higher compared to the blank control and DMSO groups (*p* < 0.001) (Figure 7E). These results suggest that isoleucine may modulate immune responses by enhancing IL-17A expression in CD4 ^+^ T cells, thereby influencing inflammation pathways.

Building upon this evidence, the subsequent study employed a murine model induced by AFB1 and HFD to establish intervention groups with graded doses of exogenous isoleucine administered via intraperitoneal injection. The study aimed to systematically evaluate the impact of isoleucine supplementation on the Th17 signaling axis, oxidative stress, intestinal barrier integrity, and hepatic injury.

### 3.8. Effects of Isoleucine on Body Weight and Organ Indices Under HFD and AFB1 Conditions

**Figure 8:**
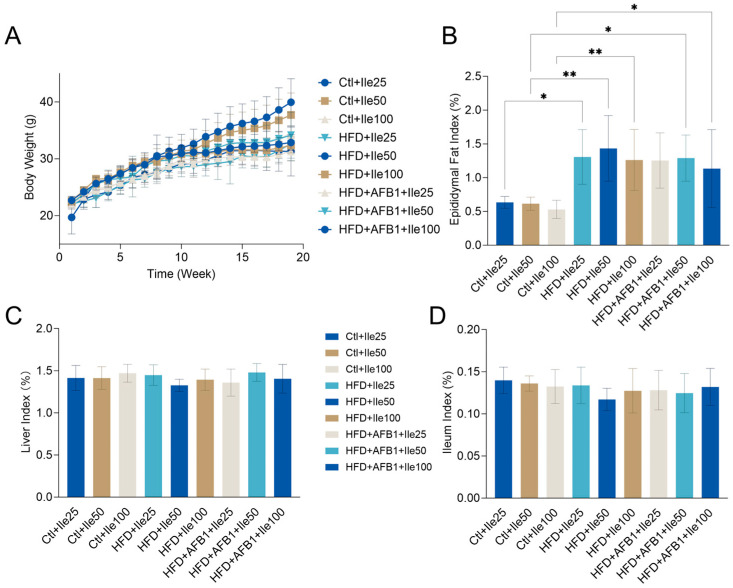
Effects of isoleucine supplementation under different metabolic contexts on body weight and organ indices in mice (n = 8, mean ± SEM). (**A**) Body weight progression over the intervention period; (**B**) Liver index (%); (**C**) Ileum index (%); (**D**) Epididymal fat index (%). Treatment groups include control diet + Ile (Ctrl + Ile25/50/100), HFD + Ile (HFD + Ile25/50/100), and HFD + AFB1 + Ile (HFD + AFB1 + Ile25/50/100). * *p* < 0.05, ** *p* < 0.01.

Figure 8 illustrates the impact of isoleucine supplementation on body weight progression and metabolic organ indices in mice subjected to varying nutritional and toxicological conditions. Under HFD conditions, the HFD + Ile50 group exhibited a significant increase in body weight, reaching a final body weight of 39.95 ± 4.14 g (*p* < 0.01 vs. Ctrl + Ile50 within the same nutritional condition), the highest body weight observed among all groups. In contrast, co-treatment with AFB1 in the HFD + AFB1 + Ile50 group significantly reduced body weight to 32.63 ± 2.94 g (*p* < 0.05 vs. HFD + Ile50 within the same HFD condition), indicating that AFB1 suppresses isoleucine-mediated weight gain under HFD conditions. Under normal diet conditions, isoleucine supplementation at various doses did not significantly alter body weight, which remained stable within the range of 31–33 g (*p* > 0.05 within normal diet groups).

No significant differences were observed in the liver index across treatment groups (range: 1.33–1.48%, *p* > 0.05), suggesting that isoleucine, HFD, and AFB1 had minimal impact on relative liver weight. Similarly, the ileum index showed no significant variation (range: 0.12–0.14%, *p* > 0.05), indicating negligible effects on intestinal tissue mass or length. For the epididymal fat index, HFD + Ile groups displayed obviously increases compared to their respective Ctrl + Ile groups within the same nutritional condition. This suggests enhanced adipose tissue accumulation under high-fat intake with isoleucine supplementation. Co-treatment with AFB1 slightly reduced the fat index, though this reduction was not statistically significant (*p* > 0.05 vs. HFD + Ile).

These findings highlight that a high-fat diet is the primary driver of obesity-related phenotypes, including body weight gain and adipose accumulation. Further supplementation with AFB1 did not result in significant changes to these parameters, which is consistent with the results shown in Figure 1.

### 3.9. Histological, Immunological, and Lipid Deposition Effects of Isoleucine Supplementation Under Different Conditions

**Figure 9:**
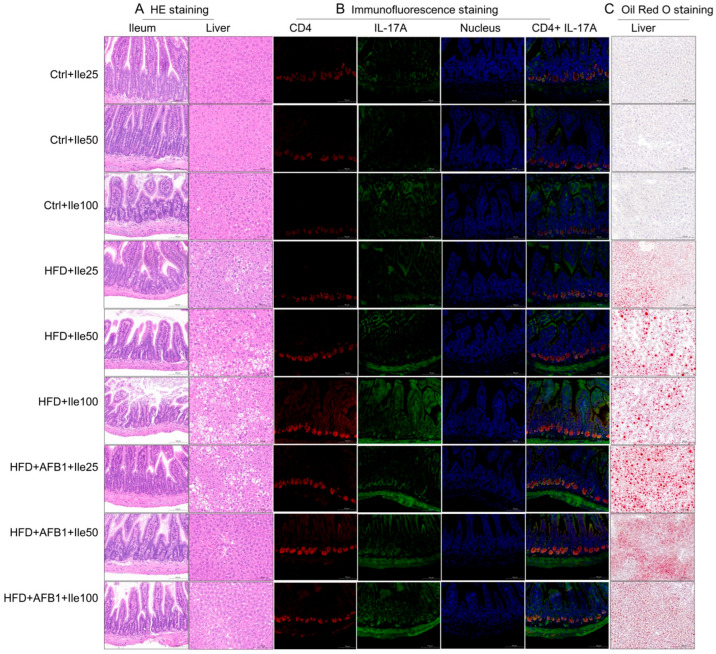
Histological, immunofluorescent, and lipid accumulation analysis of the ileum or/and liver tissues from mice treated with different doses of isoleucine under normal diet, HFD, or combined HFD and AFB1 exposure. (**A**) H&E staining of ileal and hepatic sections; (**B**) Immunofluorescence staining for CD4^+^ and IL-17A^+^ expression in ileal tissues (co-localization shown in merged panels); (**C**) Oil Red O staining indicating hepatic lipid droplet deposition. Treatment groups include control diet with 25/50/100 mg/kg Ile, HFD with isoleucine, and HFD + AFB1 with isoleucine. All the scale bars were 100 µm.

As shown in Figure 9, H&E staining revealed that liver tissues in the control groups (Ctrl + Ile 25/50/100 mg) exhibited normal morphology, with uniform cytoplasm, no vacuolization, and no pathological alterations. Colonic mucosa showed intact villus architecture, well-defined crypts, and normal goblet cell distribution. In contrast, the HFD + Ile groups displayed varying degrees of hepatic cell swelling, cytoplasmic pallor, lipid droplet vacuolization, and cellular lysis, with severity increasing in a dose-dependent manner and peaking in the HFD + Ile100 group. In the ileum, villus shortening, crypt elongation, and reduced goblet cell numbers were noted across all HFD groups. In the combined HFD + AFB1 + Ile groups, despite severe hepatocellular vacuolation and lysis at 25 mg isoleucine, hepatic architecture showed noticeable recovery at 50 and 100 mg doses, suggesting a protective effect at moderate and high doses. However, the colonic structure remained disorganized, indicating that this protective effect did not extend to the intestinal level.

Immunofluorescence analysis further elucidated the activation of the Th17 immune axis in ileal tissues. In the control groups, CD4^+^ and IL-17A^+^ cells were sparse, with weak fluorescent signals and minimal colocalization. In the HFD + Ile groups, fluorescent intensity increased in a dose-dependent manner, with the 100 mg group showing the strongest co-expression of CD4 and IL-17A, indicating that isoleucine exacerbates Th17 activation in a lipid-rich environment. In the combined treatment groups, no enhancement was observed at 25 mg isoleucine, but significant colocalization and intensified fluorescence emerged at 50 mg. However, no further increase was observed at 100 mg, suggesting a potential plateau or saturation in immune activation.

Oil Red O staining revealed hepatic lipid accumulation patterns. No visible lipid droplets were detected in Ctrl + Ile groups, indicating preserved hepatic lipid homeostasis. In contrast, HFD + Ile groups exhibited prominent red lipid droplets with increased size and density, indicating that isoleucine did not mitigate HFD-induced steatosis. Notably, in the combined HFD + AFB1 groups, the 25 mg isoleucine group showed extensive lipid accumulation, while the 50 and 100 mg groups displayed markedly smaller droplet size despite persistent density, suggesting that isoleucine may modulate lipid metabolism under toxic stress conditions.

In summary, isoleucine demonstrated a dual modulatory effect under combined HFD and AFB1 exposure. While mid- and high-dose isoleucine exacerbated hepatic steatosis and Th17 inflammation under HFD conditions, it conferred partial protection against lipid accumulation and hepatic structural damage in the presence of AFB1.

### 3.10. Modulatory Effects of Isoleucine Under Different Nutritional and Toxicological Contexts

**Figure 10:**
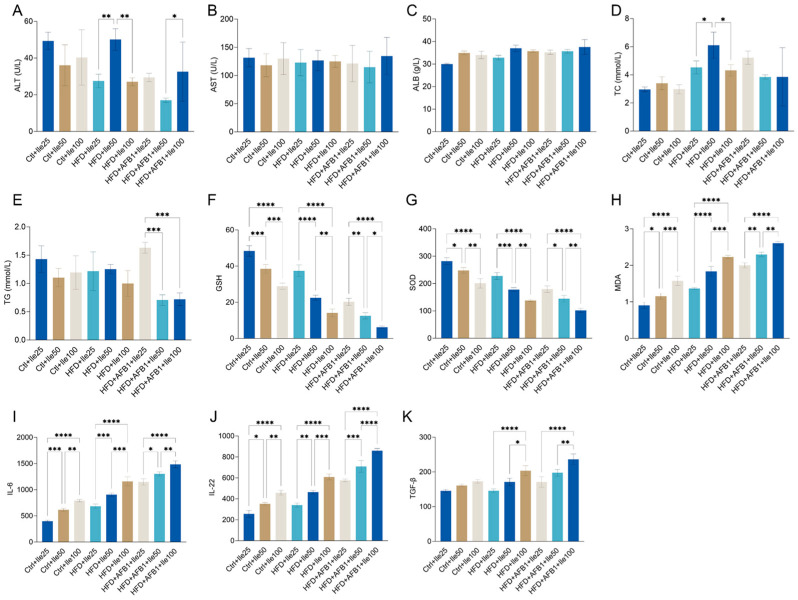
Effects of different doses of isoleucine (Ile: 25, 50, 100 mg/kg) combined with HFD and AFB1 on serum biochemical parameters in mice (n = 3 per group, mean ± SD). (**A**) ALT, U/L; (**B**) AST, U/L; (**C**) ALB, g/L; (**D**) TC, mmol/L; (**E**) TG, mmol/L; (**F**) GSH, µmol/g protein; (**G**) SOD, U/mg protein; (**H**) MDA, nmol/g protein; (**I**) IL-6, pg/mL; (**J**) IL-22, pg/mL; (**K**) TGF-β, pg/mL. Statistical significance is indicated only for comparisons between isoleucine doses under the same dietary condition. * *p* < 0.05, ** *p* < 0.01, *** *p* < 0.001, and **** *p* < 0.0001.

To examine the dose-dependent effects of isoleucine on liver function and lipid metabolism under varying dietary and toxicological conditions, serum biochemical parameters were assessed in nine groups (Figure 10A–E). Under HFD conditions, a significant increase in ALT was observed in the 50 mg/kg isoleucine group compared to both 25 and 100 mg/kg groups (*p* < 0.01), while no significant dose-dependent differences were detected in AST levels across the HFD + Ile groups. In contrast, under HFD + AFB1 conditions, ALT levels in the 50 mg/kg Ile group were significantly lower than those in the 100 mg/kg group (*p* < 0.05), with no significant changes in AST levels among doses.

ALB levels remained consistent (30–38 g/L) across all groups, with no significant intragroup differences. Regarding lipid metabolism, TC levels were significantly higher in the HFD + Ile50 compared to Ile25 and Ile100 groups (*p* < 0.05), suggesting a dose-responsive increase. Conversel, no significant dose-dependent differences in TC were observed in the Ctrl or HFD + AFB1 backgrounds. For TG, significant reductions were observed in the HFD + AFB1 + Ile50 and Ile100 groups compared to the HFD + AFB1 + Ile25 group (*p* < 0.001), while no significant changes were found within either the Ctrl or HFD groups.

As illustrated in Figure 10F–H, isoleucine supplementation exerted dose-dependent effects on oxidative stress markers under all dietary backgrounds. In the control group, increasing isoleucine doses significantly reduced GSH and SOD levels while elevating MDA levels, indicating impaired antioxidant capacity and enhanced lipid peroxidation. Similar patterns were observed under HFD conditions, where GSH and SOD were markedly depleted in the Ile50 and Ile100 groups, and MDA levels significantly increased. Under combined HFD and AFB1 exposure, isoleucine supplementation further exacerbated oxidative imbalance: GSH and SOD were significantly reduced at higher doses, while MDA accumulation was notably enhanced. Collectively, these findings suggest that under normal, HFD and AFB1 stress conditions, isoleucine induces oxidative stress in a dose-responsive manner.

In Figure 10I–K, under normal chow conditions, isoleucine also demonstrated a clear dose-dependent effect, with 50 and 100 mg/kg treatments increasing IL-6 levels to 616.14 ± 29.48 pg/mL (*p* < 0.001) and 791.81 ± 24.43 pg/mL (*p* < 0.0001), respectively. Under HFD conditions, even a low dose of isoleucine (25 mg/kg) significantly elevated IL-6 expression (683.76 ± 41.78 pg/mL, *p* < 0.01). Notably, IL-6 levels surged further to 1303.60 ± 38.67 pg/mL in the HFD + AFB1 + Ile50 group (*p* < 0.0001), indicating a strong synergistic proinflammatory effect under triple exposure. A similar pattern was observed for IL-22 and TGF-β, particularly in the co-treatment groups. In the HFD + AFB1 + Ile100 group, IL-22 levels reached 859.87 ± 21.94 pg/mL and TGF-β levels increased to 236.44 ± 15.26 pg/mL-representing respective increases of 236% and 62% compared to the Ctrl + Ile25 group. These findings suggest that medium and high doses of Ile, particularly in the presence of HFD and AFB1, significantly upregulate serum proinflammatory and immunoregulatory cytokines. This implies a potential immunostimulatory risk associated with isoleucine supplementation under metabolically stressful conditions.

## 4. Discussion

AFB1 and HFD are recognized as typical toxicological and nutritional risk factors in modern dietary patterns [11]. Using a murine model, we systematically assessed the individual and combined effects of AFB1 and HFD, along with graded isoleucine supplementation, across multiple physiological domains including metabolic parameters, hepatic function, inflammatory responses, oxidative stress markers, and gut microbiota composition. The results reveal distinct interaction patterns and underlying mechanisms of these factors under varying nutritional and toxicological conditions.

### 4.1. HFD as a Primary Stressor Aggravated by AFB1 and Isoleucine

Our findings confirm that HFD acts as the predominant driver of body weight gain and hepatic enzyme elevation in mice, indicative of early-stage metabolic liver dysfunction [24]. Under this metabolic burden, moderate-dose isoleucine supplementation (50 mg/kg) further aggravated immunometabolic imbalance, as evidenced by elevated proinflammatory cytokines (IL-6, IL-17), depletion of antioxidant defenses (GSH, SOD), and increased lipid peroxidation (MDA), constituting a characteristic “metabolic–inflammatory phenotype” [25]. Interestingly, under combined HFD and AFB1 conditions, moderate-to-high dose isoleucine supplementation (50–100 mg/kg) demonstrated a protective effect against hepatic structural damage and reduced lipid droplet size compared to low-dose supplementation (25 mg/kg), though it did not significantly affect TC levels. These results highlight the dose- and context-dependent plasticity of isoleucine’s metabolic effects, particularly under toxicological stress.

### 4.2. Th17 Axis Activation and Oxidative Stress Cooperatively Disrupt Hepatic Homeostasis

In the initial phase, flow cytometry and immunofluorescence analyses revealed significantly increased CD4^+^IL-17A^+^ T cells in both HFD and AFB1 groups, with the highest Th17 activation in the HFD + AFB1 group. This confirmed that AFB1 and HFD independently promote Th17 expansion, and their co-exposure has a synergistic effect on Th17-mediated inflammation. In the intervention phase, isoleucine supplementation showed context-dependent effects. Under HFD, mid- and high-dose isoleucine (50–100 mg/kg) exacerbated hepatic steatosis and Th17-mediated inflammation. In contrast, under combined HFD + AFB1 exposure, isoleucine offered partial protection against liver damage despite persistent lipid accumulation, with the highest proinflammatory cytokines observed in the HFD + AFB1 + Ile50 group.

The inflammatory changes paralleled oxidative stress markers. In the initial phase, HFD + AFB1 significantly increased oxidative stress compared to individual exposures. In the intervention phase, isoleucine supplementation reduced antioxidant capacity and increased lipid peroxidation, especially under HFD conditions.

These findings highlight that isoleucine’s effect on hepatic homeostasis depends on the metabolic and toxicological environment. Our data show that isoleucine exacerbates inflammation and oxidative damage under metabolic overload (HFD), consistent with Yu’s study [26], while partially protecting against liver injury in the presence of AFB1. This context-dependent duality is crucial for understanding amino acid supplementation’s therapeutic potential in metabolic disorders.

### 4.3. Microbiota Dysbiosis, Barrier Disruption, and Gut–Liver Axis Perturbation

16S rRNA sequencing revealed that co-exposure to AFB1 and HFD significantly reduced α-diversity and enriched proinflammatory genera such as *Bilophila* and *Oscillospira*, while depleting beneficial commensals such as *Akkermansia*-especially in the AFB1 + HFD group. This microbial imbalance was accompanied by decreased expression of tight junction proteins ZO-1 and Occludin, indicating compromised intestinal barrier integrity. These changes coincided with increased inflammatory cytokines and oxidative markers, suggesting a self-reinforcing cycle involving dysbiosis, barrier disruption, and systemic inflammation. Previous studies have shown that both AFB1 and HFD independently impair gut microbial composition, increase intestinal permeability, and promote systemic inflammation via LPS-mediated activation of the TLR4–NF-κB axis [11,27,28,29]. Fecal microbiota transplantation (FMT) from AFB1-treated mice has further demonstrated a causal role of dysbiosis in triggering intestinal barrier impairment and hepatic inflammation in recipient animals [9,30]. These findings collectively support the existence of a synergistic “microbiota–barrier–inflammation” cascade underlying AFB1 and HFD-induced gut–liver axis dysfunction.

### 4.4. Mechanistic Implications of Dose–Environment Dependency of Isoleucine

Under normal dietary conditions, isoleucine induced dose-dependent increases in inflammatory markers and oxidative stress despite minimal metabolic changes. In HFD contexts, moderate-dose isoleucine (50 mg/kg) specifically exacerbated hepatic injury, as evidenced by peak ALT elevation compared to lower and higher doses. Notably, under combined HFD and AFB1 exposure, isoleucine exhibited a dual modulatory pattern: while 50 mg/kg intensified inflammatory responses (IL-6: 1303.60 pg/mL, *p* < 0.0001), higher doses (100 mg/kg) reduced triglycerides compared to 50 mg/kg, suggesting context-dependent activation of protective mechanisms. Histological analyses corroborated these findings, revealing hepatic structural recovery at 50–100 mg/kg doses despite persistent intestinal damage. Previous study indicates that HFD amplifies amino acid-induced metabolic dysregulation, while AFB1 exposure enhances oxidative stress and hepatotoxicity, which may trigger endogenous protective mechanisms [10,31]. Amino acid supplementation, including threonine and branched-chain amino acids, has exhibited dose- and context-dependent effects in metabolic stress models, reinforcing the importance of tailored nutritional strategies [31,32].

Although simultaneous exposure to HFD, AFB1, and isoleucine is rare in real-life scenarios, the dose-dependent dual modulation mechanism of isoleucine under the combined stress of “metabolic dysregulation (HFD)-exogenous toxin (AFB1)” revealed in this study provides crucial experimental evidence for understanding the role of amino acids under multiple pathophysiological conditions. This finding is especially valuable for designing targeted nutritional intervention strategies for high-risk populations with metabolic abnormalities and potential toxin exposure (e.g., certain occupational groups or individuals with unbalanced diets and exposure to AFB1-contaminated environments), as well as for exploring the “double-edged sword” effect of branched-chain amino acids in liver injury progression.

## 5. Conclusions

This study demonstrates that HFD serves as the primary metabolic disruptor, while low-dose AFB1 alone exerts minimal effects. Their combination, however, synergistically disrupts gut–liver axis homeostasis through intestinal barrier impairment, oxidative stress amplification, and Th17-mediated inflammation. Gut microbiota analysis confirms HFD as the dominant driver of dysbiosis, with AFB1 + HFD intensifying the depletion of protective taxa like *Akkermansia*. Metabolomic and functional validation identified isoleucine as a key inflammatory biomarker that promotes IL-17A^+^CD4^+^ T cell differentiation. Crucially, isoleucine exhibits context-dependent duality: exacerbating pathology under HFD conditions while paradoxically attenuating hepatic lipid accumulation at moderate/high doses under AFB1 + HFD exposure. These findings establish that isoleucine functions as a double-edged sword—amplifying metabolic inflammation in lipid-rich environments while potentially activating compensatory pathways under combined toxicant exposure. The results underscore the critical importance of environmental context in determining functional amino acid effects, necessitating condition-specific safety evaluations for nutritional interventions in metabolically compromised individuals.

## Data Availability

The data presented in this study are available on request from the corresponding author due to privacy.

## Abbreviations

The following abbreviations are used in this manuscript:

AFB1	Aflatoxin B1
ALB	Albumin
ALT	Alanine Aminotransferase
ANOVA	Analysis of Variance
AST	Aspartate Aminotransferase
BCAAs	Branched-Chain Amino Acids
BSH	Bile Salt Hydrolase
DMSO	Dimethyl Sulfoxide
GSH	Glutathione
H&E	Hematoxylin and Eosin
HFD	High-Fat Diet
Ile	Isoleucine
LEfSe	Linear Discriminant Analysis Effect Size
MDA	Malondialdehyde
NAFLD	Non-Alcoholic Fatty Liver Disease
OTU	Operational Taxonomic Unit
SPF	Specific Pathogen-Free
TG	Triglycerides
VLDL	Very-Low-Density Lipoprotein
